# Prevalence of Islet Autoantibodies in Adults Without Diabetes

**DOI:** 10.1210/jendso/bvaf095

**Published:** 2025-05-20

**Authors:** Meghan E Pauley, Kimber M Simmons, Fran Dong, Liping Yu, Andrea K Steck, Cristy Geno Rasmussen, Brigitte I Frohnert, Marian J Rewers

**Affiliations:** Barbara Davis Center for Diabetes, School of Medicine, University of Colorado Anschutz Medical Campus, Aurora, CO 80045, USA; Barbara Davis Center for Diabetes, School of Medicine, University of Colorado Anschutz Medical Campus, Aurora, CO 80045, USA; Barbara Davis Center for Diabetes, School of Medicine, University of Colorado Anschutz Medical Campus, Aurora, CO 80045, USA; Barbara Davis Center for Diabetes, School of Medicine, University of Colorado Anschutz Medical Campus, Aurora, CO 80045, USA; Barbara Davis Center for Diabetes, School of Medicine, University of Colorado Anschutz Medical Campus, Aurora, CO 80045, USA; Barbara Davis Center for Diabetes, School of Medicine, University of Colorado Anschutz Medical Campus, Aurora, CO 80045, USA; Barbara Davis Center for Diabetes, School of Medicine, University of Colorado Anschutz Medical Campus, Aurora, CO 80045, USA; Barbara Davis Center for Diabetes, School of Medicine, University of Colorado Anschutz Medical Campus, Aurora, CO 80045, USA

**Keywords:** type 1 diabetes, adult-onset diabetes, LADA, latent autoimmune diabetes in adults, autoimmune diabetes, islet autoantibodies

## Abstract

**Context:**

Over half of all new cases of type 1 diabetes (T1D) are diagnosed in adults, yet the natural history of adult-onset T1D, particularly in nonfamilial populations, is not fully understood.

**Objective:**

This study measured the prevalence of islet autoantibodies (IA) in adults without known diabetes and irrespective of T1D family history from Colorado (USA).

**Methods:**

The Autoimmunity Screening for Kids study screened for IAs to insulin, glutamic acid decarboxylase (GADA), islet antigen-2, and zinc transporter 8 in 1087 adults without known diabetes [mean age 40.7 years with range 19.6-63.9 years, 63% non-Hispanic White (NHW), 10% with family history of T1D in a first-degree relative, and 78% female] from Colorado. IAs were measured using radiobinding assay and electrochemiluminescence detection methods.

**Results:**

In total, 3.86% of adults screened positive for any IA, 0.55% screened positive for multiple IAs, and 1.75% were positive for a single IA by both detection methods. Compared to NHW, those with Hispanic race/ethnicity were more likely to screen positive for a single IA (relative risk 2.32, 95% confidence interval 1.40, 3.84, *P* = .001), but there was no difference in the risk of screening positive for multiple IAs when comparing across race/ethnicity. GADA was the most prevalent IA, found in 2.67% of adults.

**Conclusion:**

IA prevalence was high in this sample of adults without known diabetes from Colorado. Further study is needed to fully characterize the risk of progression to clinical diabetes among adults who screen positive for IAs, particularly in nonfamilial populations.

Traditionally, type 1 diabetes (T1D) has been regarded as a disease primarily impacting children and adolescents. Yet numerically, more adults than youth are being diagnosed with new-onset T1D. In 2021, there were an estimated 510 000 new cases of T1D diagnosed globally, and approximately 62% of those were in adults aged 20 years and older [1]. Development of islet autoantibodies (IA) precedes the onset of clinical disease, and IA screening studies show that approximately 70% of youth who screen positive for multiple IAs progress to clinical (stage 3) T1D within 10 years, while a single positive IA confers a lower risk [2, 3]. However, the natural history of T1D is heterogeneous across the lifespan [4]. Adults with incident T1D are believed to demonstrate slower disease progression [5-7] and slower loss of β-cell function after diagnosis, compared to youth [8, 9]. Longitudinal studies in youth and in adults with a genetic risk of T1D and/or family history of T1D have been instrumental to our current understanding of the pathogenesis of T1D, yet there remain significant gaps in understanding the prevalence of IAs in adults and the associated risk of progression to clinical diabetes among adults from the general population [10]. The goal of this study was to explore the prevalence of multiple or single IAs in adults without known diabetes and irrespective of T1D family history.

## Materials and Methods

### Study Population

This study was approved by the Colorado Multiple Institutional Review Board. The Autoimmunity Screening for Kids (ASK) study has conducted population-based screening for T1D IAs and celiac disease transglutaminase autoantibodies in youth ages 1 to <18 years old since 2017 [11]. There was no cost for participation in the study. At the time of this study in adults, Colorado residency and English or Spanish language were required for enrollment, and participants were recruited from the Denver, Colorado, metropolitan area. Recruitment strategies included a combination of approaches, such as paper materials (flyers/posters) displayed in clinics, hospitals, and community settings; advertising booths at community events; awareness initiatives to inform local healthcare providers (primary care physicians, endocrinologists) about the ASK study; and ASK study team members recruiting, enrolling, and conducting screening in healthcare settings. Upon enrollment, self-report of demographic information, personal medical history surrounding diabetes and celiac disease, and family history of T1D and celiac disease in first-degree relatives was recorded. This study defines a T1D first-degree relative (FDR) as a parent, child, or sibling of a person with T1D.

This study population consisted of 1087 adult parents/guardians who were screened for IAs between February 2021 and February 2022 as part of the ASK study, while their children were also enrolled to undergo screening in the ASK study. At the time of enrollment, participants were not selected based on family history of diabetes. While some individuals did report a family history of T1D, this was not an exclusion criterion. Anyone reporting an existing diabetes diagnosis was excluded from enrollment, but those with a self-reported celiac disease diagnosis were permitted to enroll.

Participants who screened positive for any IAs with either or both of the detection methods described here were invited for a confirmation visit within 3 months of screening. If IA results were confirmed positive for any IA, participants were invited to participate in a longitudinal monitoring research program; ASK monitoring program methods are aligned with recently published consensus guidelines [12]. This analysis focuses largely on initial IA screening results.

### Autoantibody Screening

Autoantibodies to insulin (IAA), glutamic acid decarboxylase (GADA), islet antigen-2 (IA-2A), and zinc transporter 8 (ZnT8A) were measured in the Immunogenetics Laboratory at the Barbara Davis Center for Diabetes by both radiobinding assay (RBA) and high-affinity electrochemiluminescence (ECL) detection [13-15] for IA screening. Initial IA screening is performed on a capillary blood sample, and then at the time of the confirmation visit, testing is repeated on venous blood obtained via venipuncture. The threshold for positivity for these assays was set at the 99th percentile of the control population. Furthermore, the 2020 Islet Autoantibody Standardization Program Workshop reported the sensitivities and specificities for RBA in incident type 1 diabetes to be 78% and 99% for GAD antibody; 72% and 100% for IA-2 antibody; 62% and 99% for insulin autoantibody; and 74% and 100% for ZnT8 antibody. Sensitivities and specificities for ECL were reported as 78% and 100% for GAD antibody, 72% and 100% for IA-2 antibody, and 66% and 99% for insulin autoantibody [16-18]. The number and type of IAs detected, as well as method(s) of detection, were recorded for each participant.

### Statistical Analyses

All analyses were performed using GraphPad Prism 8.4 Software and SAS v9.4 (SAS Institute Inc., Cary, NC). Demographic characteristics and IA prevalence are presented as mean (SD) for continuous variables and percentages for categorical variables. Poisson regression with robust error variance was used to examine associations between covariates including race/ethnicity, FDR status, and sex and IA screening results [defined as screening positive for multiple IAs (multiple IA+), screening positive for a single IA by both RBA and ECL detection methods (single IA+ by both methods), or screening positive for a single IA by only 1 detection method (single IA+ by 1 method)]. Relative risk (RR) and 95% confidence intervals (CIs) were evaluated.

Poisson regression with robust error variance and chi-square statistical tests were also used to compare adult screening results to a selection (n = 7595) of ASK study youth who were recruited from the same population. The largest possible sample, frequency matched to the adult participants on race/ethnicity, sex, and T1D FDR status, was selected. This selected sample of youth participants was enrolled in the ASK study as part of general-population IA screening, screened between January 2017 and February 2022.

## Results

### Baseline Characteristics and IA Prevalence

A total of 1087 adults completed screening. Ages ranged from 19.6 to 63.9 years with a mean age of 40.7 years. Participant characteristics and IA screening results are reported in Table 1. Overall, 42 screened positive for at least 1 IA. Of those, 6 screened multiple IA+, 19 screened single IA+ by both methods, and 17 screened single IA+ by 1 method. Of those 17 who screened single IA+ by 1 method, 16 were detected by RBA and 1 was detected by ECL. GADA was the most common IA found in all 3 groups: multiple IA+, single IA+ by both methods, and single IA+ by 1 method. Table 2 reports IA screening results stratified by decade of age. The majority of participants, including the majority of those who screened IA+, were between 30 and 39.9 years (41.86%) and 40 and 49.9 years of age (43.24%). Of note, 30 adults reported a personal history of celiac disease, and all 30 screened negative for any IAs.

**Table 1.** Participant characteristics at screening

	All participants (n = 1087)	Multiple IA+ (n = 6)	Single IA+ by both methods (n = 19)	Single IA+ by 1 method (n = 17)	IA negative (n = 1045)
Age in years, mean ± SD (range)	40.7 ± 6.9 (19.6-63.9)	43.1 ± 8.0 (32.1-54.4)	40.8 ± 4.9 (30.4-48.6)	39.9 ± 6.0 (25.4-49.3)	40.7 ± 6.9 (19.6-63.9)
Sex, n (%)					
Female	844 (77.6)	5 (83.3)	15 (78.9)	15 (88.2)	809 (77.4)
Male	243 (22.4)	1 (1.7)	4 (21.1)	2 (11.8)	236 (22.6)
Race/ethnicity, n (%)					
Non-Hispanic White	684 (62.9)	4 (66.7)	5 (26.3)	9 (52.9)	666 (63.7)
Hispanic, any race	296 (27.2)	1 (16.7)	12 (63.2)	7 (41.2)	276 (26.4)
Other race, not Hispanic	107 (9.8)	1 (16.7)	2 (10.5)	1 (5.9)	103 (9.9)
T1D FDR, n (%)	112 (10.3)	2 (33.3)	3 (15.8)	1 (5.9)	106 (10.1)
Positive IAs, n	—	GADA + IAA: 2 GADA + ZnT8A: 2 ZnT8A + IA-2A: 1 GADA + ZnT8A + IAA: 1	GADA: 13 IAA: 5 ZnT8A: 1 IA-2A: 0	GADA: 11 IAA: 5 ZnT8A: 1 IA-2A: 0	—

Abbreviations: FDR, first-degree relative; GADA, glutamic acid decarboxylase; IA, islet autoantibody; IAA, insulin; IA-2A, islet antigen-2; T1D, type 1 diabetes; ZnT8A, zinc transporter 8.

**Table 2.** Islet autoantibody results by decade of age

Age, years	All participants (n = 1087)	Multiple IA+ (n = 6)	Single IA+ by both methods (n = 19)	Single IA+ by 1 method (n = 17)	IA negative (n = 1045)
19-29.9	58 (5.3)	0 (0.0)	0 (0.0)	2 (11.8)	56 (5.4)
30-39.9	455 (41.9)	2 (33.3)	8 (42.1)	6 (35.3)	439 (42.0)
40-49.9	470 (43.2)	3 (50.0)	11 (57.9)	9 (52.9)	447 (42.8)
50-59.9	100 (9.2)	1 (16.7)	0 (0.0)	0 (0.0)	99 (9.5)
60-69.9	4 (0.4)	0 (0.0)	0 (0.0)	0 (0.0)	4 (0.4)

Results reported as number (%).

Abbreviations: IA, islet autoantibody.

**Figure 1.**
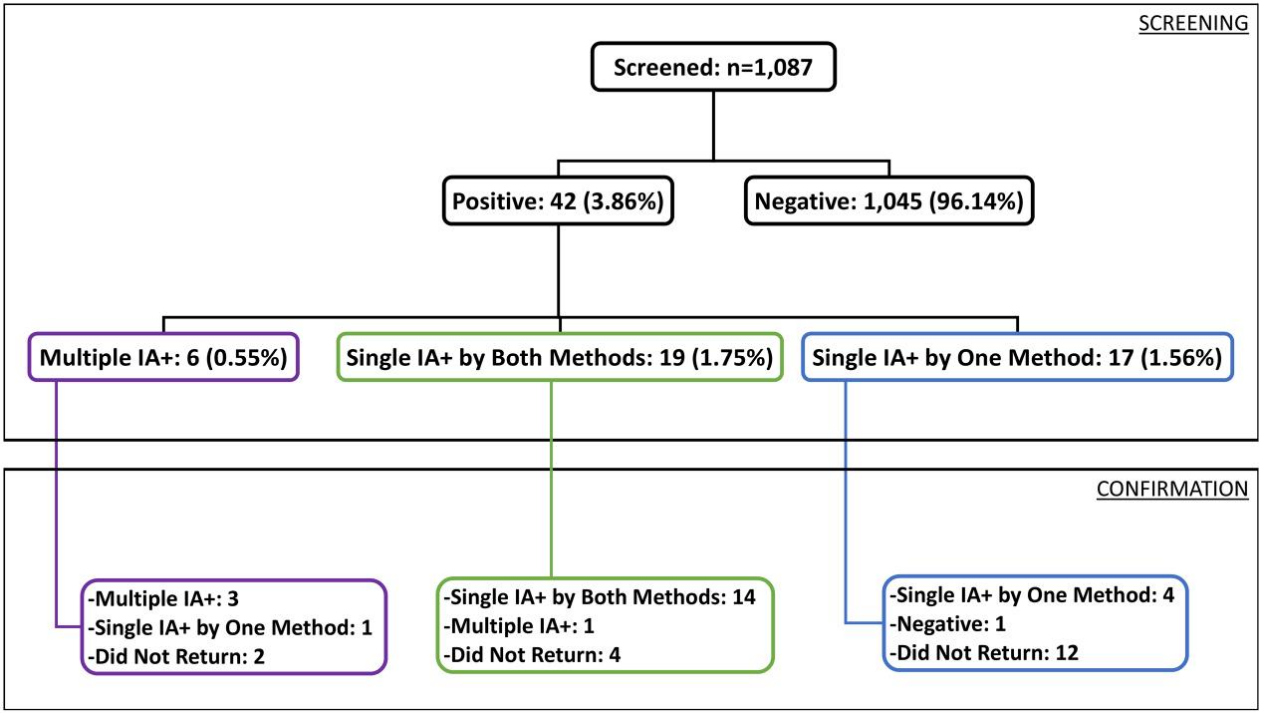
IA screening and confirmation results. The figure depicts a flow diagram reporting the results of screening and confirmation IA testing in 1087 screened adults. The larger top black box depicts screening results, first stratified by negative or any positive result. Positive results are then stratified by degree of IA positivity [multiple islet autoantibody positive (multiple IA+), single islet autoantibody positive by both RBA and ECL detection methods (single IA+ by both methods), and single islet autoantibody positive by either RBA or ECL detection method (single IA+ by 1 method)]. The smaller bottom black box depicts results from the 24 participants who returned for confirmation testing, grouped by IA status at the time of initial screening, to show any discordance between screening and confirmation results. Abbreviations: ECL, electrochemiluminescence; IA, islet autoantibody; RBA, radiobinding assay.

Figure 1 depicts a flowchart reporting results from both initial screening and subsequent confirmation testing. Of the 42 participants who screened positive for any IA, 24 completed confirmation testing. IA+ status was confirmed in all but 1 adult. The rate of participation in confirmation testing was higher in adults with multiple IA+ or single IA+ by both methods compared to those with single IA+ by 1 method.

### Comparison of Adults and Frequency-matched Youth

To test the hypothesis that the prevalence of IA+ is lower in adults compared to youth of comparable race/ethnicity, FDR status, and sex, the largest possible sample of ASK study youth was frequency matched to the adult participants on race/ethnicity (*P* = .35), T1D FDR status (*P* = .87), and sex (*P* = .98). This youth sample (n = 7595) had a mean age of 9.3 years old (range 1.0-18.0 years), was 62.86% NHW, included 10.14% FDRs, and was 77.60% female. The prevalence of multiple IA+ was not statistically different across the adult and youth cohorts (0.55% of adults compared to 0.53% of youth, *P* = .91); however, the prevalence of single IA+ by both methods was higher in adults (1.75% of adults compared to 0.59% of youth, *P* < .001). The prevalence of single IA+ by 1 method was also similar across groups (1.56% of adults compared to 1.97% of youth, *P* = .36). This data is not presented in a table or figure.

### Relationships Among Patient Characteristics and IA Prevalence

**Figure 2.**
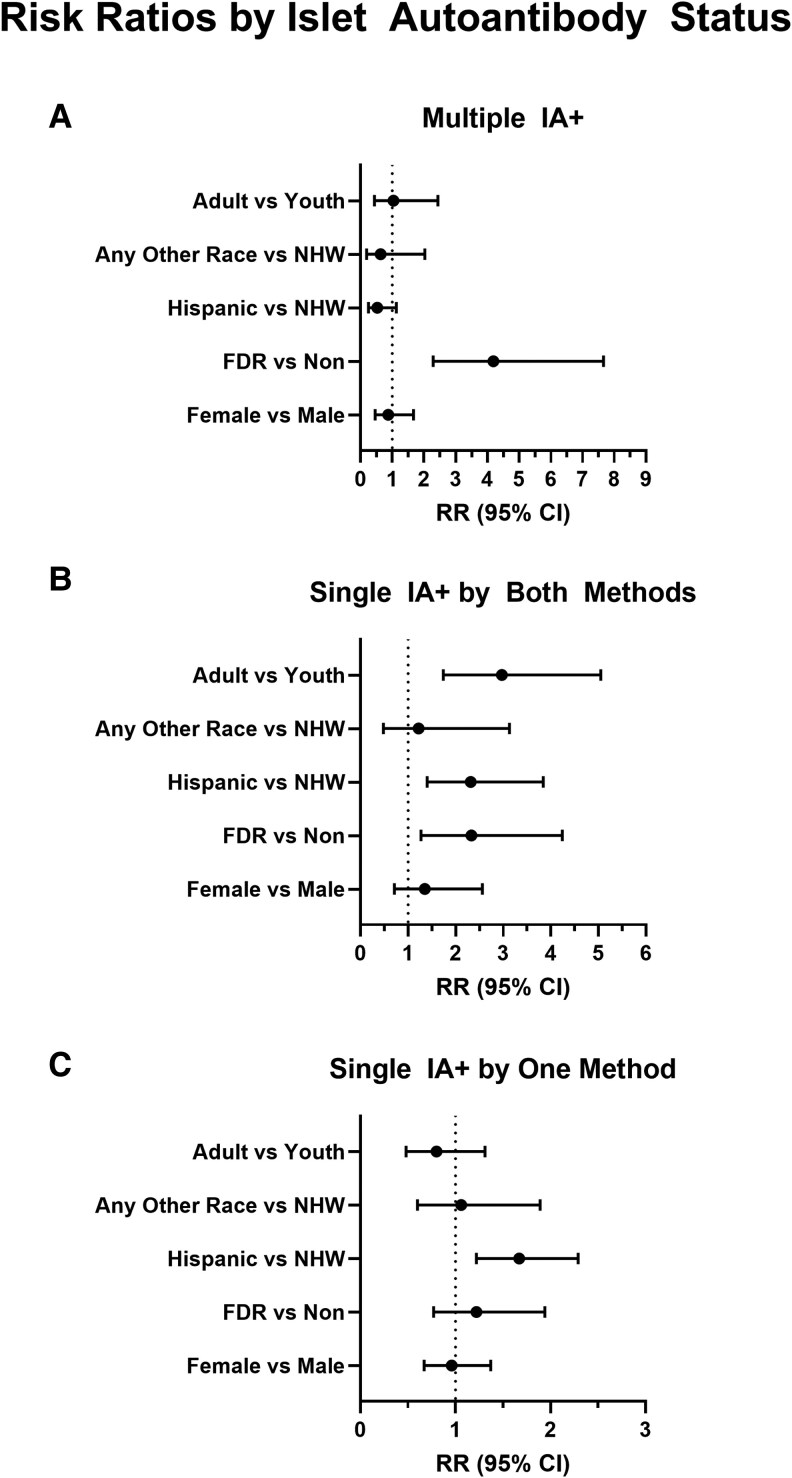
Risk ratios for demographic characteristics by IA positivity status. Figures depict risk ratios (dots) with 95% confidence intervals (bars) for each IA positivity status group in relation to patient characteristics: (A) multiple islet autoantibody positive (multiple IA+); (B) single islet autoantibody positive by both RBA and ECL detection methods (single IA+ by both methods); (C) single islet autoantibody positive by either RBA or ECL detection method (single IA+ by 1 method). X-axes depict risk ratios in linear scale. Abbreviations: ECL, electrochemiluminescence; FDR, first-degree relative; IA, islet autoantibody; NHW, non-Hispanic White; Non, nonrelative; RBA, radiobinding assay.

Results of Poisson regression models are summarized in Fig. 2. Compared to youth, adults were more likely to screen single IA+ by both methods (RR 2.97, 95% CI 1.74, 5.05, *P* < .001). However, the risk of screening both multiple IA+ and single IA+ by 1 method were similar in adults and youth (RR 1.04, 95% CI .44, 2.44, *P* = .93 and RR 0.80, 95% CI .48, 1.31, *P* = .37, respectively). Race/ethnicity had no effect on the likelihood of screening multiple IA+ (Fig. 2); however, those with Hispanic race/ethnicity were more likely to screen single IA+ by both methods (RR 2.32, 95% CI 1.4, 3.84, *P* = .001) and single IA+ by 1 method (RR 1.67, 95% CI 1.22, 2.29, *P* = .001) when compared to NHW adults.

As expected, FDRs were more likely to screen multiple IA+ (RR 4.19, 95% CI 2.29, 7.66, *P* < .001) or single IA+ by both methods (RR 2.33, 95% CI 1.27, 4.24, *P* = .01), compared to nonrelatives. Yet, FDR status did not impact the likelihood of screening single IA+ by 1 method (RR 1.22, 95% CI .77, 1.94, *P* = .40). No differences were seen across female vs male sex (Fig. 2).

### Progression to Clinical T1D

At the time of writing, 2 study participants were subsequently diagnosed with clinical T1D. One was a 42.9-year-old female with Hispanic race/ethnicity, single IA+ (GADA) by both methods, who did not report a family history of T1D in FDRs. Time from screening to T1D diagnosis was 19 months. The second was a 48.0-year-old female with NHW race/ethnicity, multiple IA+ (GADA, ZnT8), who also did not report a family history of T1D in FDRs. Time from ASK screening to T1D diagnosis was 10 months.

## Discussion

The ASK study screened 1087 adults without known diabetes and irrespective of T1D family history, from Colorado to assess the prevalence of T1D IAs. Results showed the prevalence of multiple IA+ in these adults was 0.55%, which was similar to that from a matched sample of Colorado youth with a similar distribution of sex, race/ethnicity, and family history of T1D in FDRs. This is a notable finding, as multiple IA+ is a defining characteristic of preclinical T1D (stage 1 or 2), and evidence from longitudinal studies in youth shows that multiple IA+ confers a near 100% lifetime risk of progression to clinical T1D [3].

Youth who screen single IA+ have an approximately 15% 10-year risk of progression to clinical T1D [3]. Furthermore, in the pediatric cohort, ASK has observed that participants who screen single IA+ by 1 method have the lowest associated risk of progression to clinical T1D, and screening single IA+ by both methods confers risk that is lower than multiple IA+ but higher than single IA+ by 1 method (unpublished data) [19, 20]. In this study, adults were more likely to screen single IA+ by both methods as compared to the matched youth cohort. However, the risk of progression to clinical T1D in IA+ adults without known genetic risk or family history of T1D is not well understood. Existing studies in relatives of those with T1D suggest that IA+ adults have slower disease progression as compared to youth or sometimes may not progress to clinical diabetes [7, 12, 21]. T1D incidence studies report a bimodal distribution of age at diagnosis, with an initial peak in childhood and a later peak in older adulthood. A study in Sweden identified the second peak as occurring between 50 and 80 years old [22], and global data suggests it occurs at around 70 years old [1]. Yet, knowledge gaps persist regarding the timing of IA positivity and the associated long-term risk of progression to clinical T1D in adults. In this study, most IA+ participants were between 30 and 49.9 years old, which is reflective of the age distribution of study participants.

T1D incidence is increasing in racial and ethnic minority populations [1]. Race and ethnicity are suggested to influence T1D phenotype, but this relationship may differ across age groups. A TrialNet study of T1D relatives found the risk of progression from single IA+ to multiple IA+ was lower in participants with Hispanic race/ethnicity compared to participants with NHW race/ethnicity, but differences in progression to clinical diabetes across race/ethnicity were influenced by body mass index and significant only in children under 12 years old [23]. It is notable that, in our study, race/ethnicity had no effect on the likelihood of screening multiple IA+, but those with Hispanic race/ethnicity were more likely to screen single IA+ when compared to NHW adults. Further study is needed to better characterize differences in the risk seen with different racial and ethnic populations across the lifespan.

Compared to nonrelatives, FDRs were more likely to screen multiple IA+ and single IA+ by both methods. This was expected, as relatives of those with T1D are shown to have an approximately 15 times higher risk of developing T1D compared to those without a family history [24, 25]. However, population-based studies prove that the majority of people who develop T1D do not have a positive family history [26], which underscores the utility of general-population screening initiatives such as this.

Interestingly, our screening results found that a positive FDR status was not associated with increased risk of screening single IA+ by 1 method. This posits the possibility of false-positive results, as screening assays rely on a defined threshold, which may not always reflect true pathology [9]. Our study utilized 2 detection methods and included additional confirmation testing to address and lessen this possibility. It is not uncommon for single positive IAs, especially when detected by RBA, to be low-affinity and associated with a lower risk of disease [13, 27-29]. Yet, single IA+ individuals can and do progress to clinical T1D, and this is associated with older age as compared to multiple IA+ progressors [30]. Furthermore, the risk of disease progression is likely increased when single IA+ is detected using high-affinity methods, such as ECL [10, 27, 28, 31, 32]. In this study, only 1 of the 17 participants who screened single IA+ by 1 method was detected by ECL, and so these participants were not differentiated into separate groups for this analysis.

Importantly, we must also consider that this finding may reflect IA fluidity across the lifespan. IA status has been shown to expand, revert from positive to negative results, and even revert then later regain IA+ [3, 5, 21, 33, 34]. Evidence suggests there are differences in the associated risk of progression across these different IA trajectories. One study found reversion from multiple IA+ to single IA+ or IA negative was associated with reduced incidence of clinical T1D and found the likeihood of reversion to be associated with older age and fewer positive IAs [33]. As our study is a cross-sectional analysis, it provides only a snapshot of the dynamic evolution of IAs, and we cannot make assertions regarding long-term risks associated with these results.

GADA was the most prevalent IA across all adults in this study, which is consistent with the existing literature in incident T1D and relative cohorts [5, 6, 35, 36]. IA characterization is 1 factor that is suggested to influence risk of progression to clinical diabetes [3, 6, 37]. Youth studies show IA-2A positivity is associated with a higher risk of IA+ expansion [38], but studies including both youth and adults demonstrate ZnT8A is associated with a higher risk of disease progression compared to any other single IA+ [31, 39]. In those who demonstrate slower progression of disease, GADA positivity is commonly the first detectable IA [40]. Indeed, a TrialNet study of relatives of people with T1D found a relationship between age and rate of disease progression that was influenced by the type of IA present [5]. The risk of progression associated with GADA has been shown to be over 4 times higher among adults 45 years of age than in youth [41].

The ASK study conducts screening for autoantibodies associated with T1D and also celiac disease, and an existing diagnosis of celiac disease does not prohibit enrollment and participation in ASK. We recognize that offering both screenings may introduce a type of selection bias, where those with a personal or family history of autoimmune disease (and therefore at higher risk of screening IA+) may be more likely to participate in this study. Yet, none of the participants with a self-reported history of celiac disease screened positive for any IAs.

Additional limitations to this study include that females were overrepresented and the majority of participants were between the ages of 30 and 49.9 years old. These findings are likely a result of ASK study recruitment methods, which included recruiting both youth and their parents/guardians simultaneously from events such as healthcare visits within primary care clinics and hospitals. We recognize that these limitations likely limit the generalizability of our results. We must also consider that the age range of these participants may be optimal for studying the natural history of adult-onset T1D, as IA detection at this age allows for future longitudinal study over decades in participants with potential for developing clinical T1D within their lifetimes. This study investigates IA prevalence and not incidence, and it is therefore unclear at what point seroconversion initially occurred in these participants. Moreover, a relatively low proportion of participants who screened IA+ returned for a confirmation visit to complete repeat testing. Lastly, although only 10% of study participants reported a family history of T1D, we recognize the potential impact of those participants on study results, and we recognize and acknowledge that this study's population is not representative of the general population.

In conclusion, the ASK study is the first multiple IA screening program to include adults without known diabetes and without a family history of T1D, addressing a significant gap in the understanding of T1D across the lifespan. Results demonstrated high IA+ prevalence in adults, similar to that seen in a matched sample of youth from the same population, as well as differences in IA+ across race/ethnicity status. Following the Food and Drug Administration’s 2022 approval of teplizumab to delay the onset of clinical T1D [42], our novel results suggest that screening adults from the general population for IAs may identify a significant number of people with T1D in preclinical stages who may benefit from therapeutic intervention to delay disease progression. Further study is needed to better characterize the long-term risks associated with IA positivity and what benefits may be seen with early detection, monitoring, and preventative intervention in adults from the general population.

## Data Availability

Data are available from the authors upon reasonable request.
